# Identifying early stages of reindeer domestication in the archaeological record: a 3D morphological investigation on forelimb bones of modern populations from Fennoscandia

**DOI:** 10.1007/s12520-020-01123-0

**Published:** 2020-07-16

**Authors:** Maxime Pelletier, Antti Kotiaho, Sirpa Niinimäki, Anna-Kaisa Salmi

**Affiliations:** 1 Archaeology, History, Culture and Communication Studies, Faculty of Humanities, University of Oulu, Oulu, Finland; 2 Department of Radiology, Oulu University Hospital, Oulu, Finland

**Keywords:** 3D geometric morphometrics, Captivity, Domestication signal, Fossil record, *Rangifer tarandus*, Zooarchaeology

## Abstract

Reindeer herding probably developed during the Late Iron Age onwards and is still an important part of the subsistence and culture of many peoples in northern Eurasia. However, despite the importance of this husbandry in the history of these Arctic people, the period and place of the origin as well as the spread of domestic reindeer is still highly debated. Besides the existence of different breeding methods in these territories, identifying domesticated individuals in the archaeological record is complicated because reindeers are considered to still be in the early phases of the domestication process. Indeed, the traditional morphological markers used in zooarchaeology to decipher the domestication syndrome are hardly perceptible in these early stages. In this work, we propose solutions for identifying domestic reindeer bones using 3D geometric morphometrics on isolated elements from the long bones of the forelimb (i.e. humerus, radio-ulna and metacarpal). These bones are important to understand both the feeding behaviour and the mobility of reindeer, and the potential effect of load-carrying or draught in the case of domestic reindeer. We analysed 123 modern specimens from Fennoscandia, including the two interbreeding subspecies currently present in these territories: mountain reindeer (*Rangifer tarandus tarandus*) and forest reindeer (*R.t. fennicus*); and where the sex and the lifestyle were known (i.e. free-ranging, racing or draught and captive individuals). A good level of discrimination between the size and shape variables of the bones of the forelimb was found among both subspecies and sexes. Moreover, individuals bred in captivity had smaller bone elements and a thinner and more slender morphology than free-ranging individuals. This demonstrates that the long bones of the forelimb can provide information on changes in feeding and locomotor behaviour prompted by the domestication process, like control and/or reduction of mobility and food of individual reindeer by humans. This also demonstrates that analysis in 3D geometric morphometrics is useful in detecting reindeer incipient domestication markers. Our results can be used by archaeologists to trace the early stages of domestication from fossil reindeer remains, and aid in reconstructing the socio-economic changes of past Arctic populations over time.

## Introduction

Reindeer (*Rangifer tarandus* Linnaeus, 1758) is the ungulate species with the largest circumpolar distribution in the northern hemisphere, extending over most of the tundra and taiga areas of Eurasia (Fennoscandia, Siberia, Mongolia) and North America (Canada, Alaska, Greenland) (Syroechkovskii 1995; Geist 1998), and whose past geographical distribution was even more important (Ukkonen et al. 2006; Banks et al. 2008; Kahlke 2014; Sommer et al. 2014). Therefore, reindeer hunting and herding have been one of the most important means of livelihood for human societies from the Palaeolithic to the present. Wild reindeer hunting was practiced in most of Southern Eurasia during the Pleistocene (Kurtén 1968; Gaudzinski and Roebroeks 2000; Grayson and Delpech 2002; Discamps et al. 2011; Kuntz and Costamagno 2011), and it continued in the Holocene until today in Arctic and boreal environments (Binford 1978; Spiess 1979; Grønnow 1986; Blehr 1990; Gordon 1990). Although some scholars estimate reindeer domestication began in the Mesolithic, it is generally supposed that reindeer herding certainly developed from the Late Iron Age (ca. 800–900 AD) onwards in Northern Fennoscandia (Helskog and Indrelid 2011; Hansen and Olsen 2014). In Siberia, dates as early as 1,500 BC have been suggested (Murashkin et al. 2016), while other researchers maintain it was much later (Ingold 1980). Nowadays, reindeer husbandry is still an important part of the subsistence, lifeways and cosmology of many peoples in Northern Eurasia (Mirov 1945; Vorren and Manker 1962; Levin and Potapov 1964; Hultkrantz 1985; Baskin 2000). In fact, approximately half of the world’s reindeer population is now considered domestic or semi-domestic (Syroechkovskii 1995; Baskin 2005). However, the exact period and the earliest hearths of domestication through archaeological records have not yet been clearly identified and are still highly debated.

**Fig. 1:**
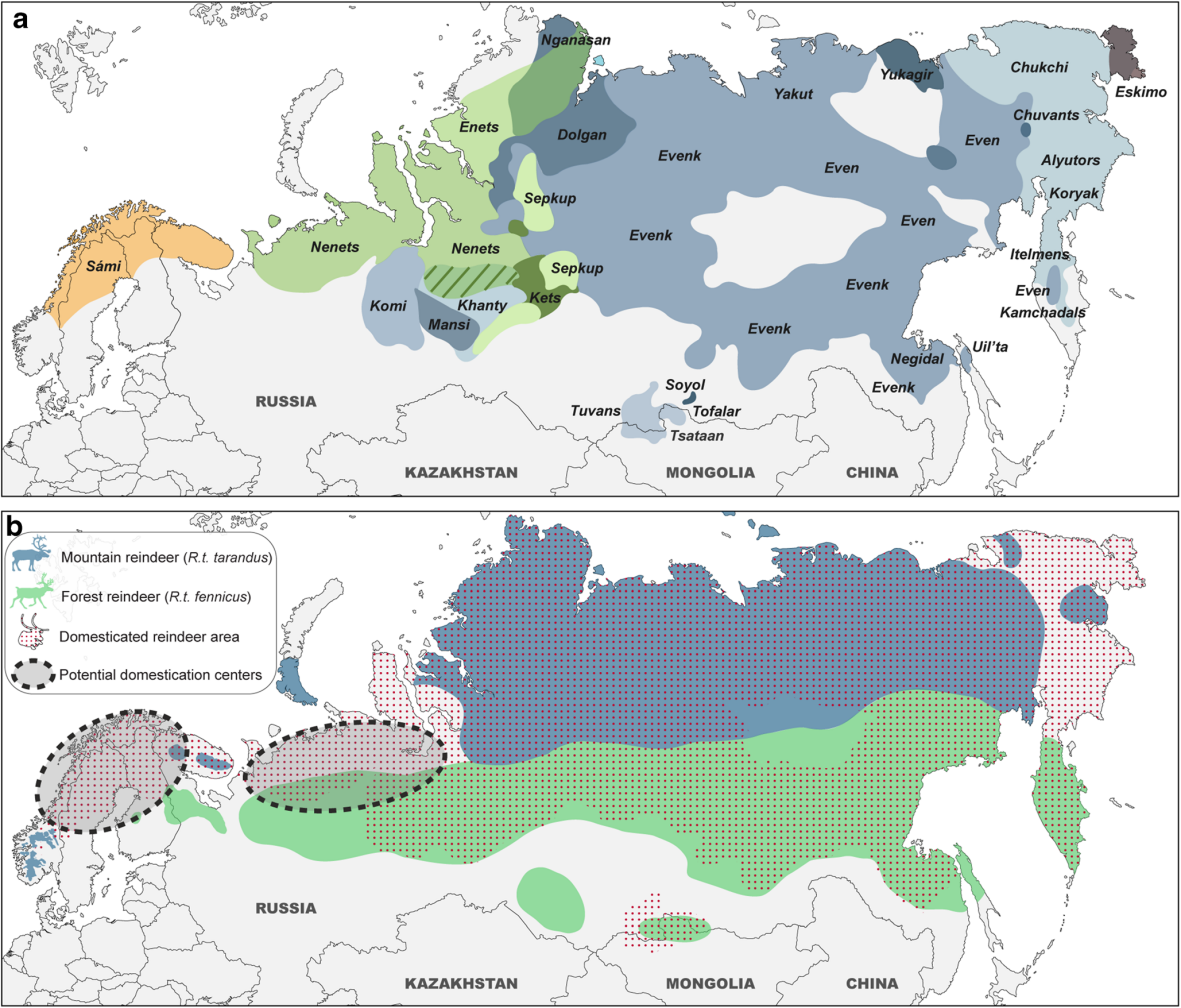
Current geographic distribution of the indigenous reindeer herders (**a**, after Kardash and Girchenko 2018; Reindeer Herding 2019) and of the two reindeer subspecies, including wild and domestic populations in Eurasia (**b**, after CAFF 2001), with localization of their potential domestication centres according to Røed et al. (2008)

Some recent genetic data show two main independent reindeer domestication poles, one in Fennoscandia and the other in Western Russia (Røed et al. 2008, 2011), corresponding to the current areas occupied by Sámi and Samoyedic populations, respectively. This implies that the Sámis would have domesticated their own reindeer independently of the indigenous cultures in northwestern Siberia. However, a recent genetic study has challenged this interpretation, showing there was evidence of a major genetic change during the 16th and 17th centuries suggesting non-native animals were introduced to northern Fennoscandia during this period, at the same time as the transition to reindeer pastoralism occurred (Røed et al. 2018). Furthermore, although many indigenous peoples have disappeared (e.g. Yughs, Mators, Kamasins, Yurats, Kereks), today there are still nearly thirty indigenous reindeer herder groups, essentially across Eurasia (Fig. 1a), with their great variability of practices and types of husbandry (Mirov 1945; Baskin 2000; Reindeer Herding 2019). Historically, most of them preserved their nomadic hunting traditions, while also practicing reindeer herding. For example, in northern Fennoscandia, Sámis used small domestic herds as decoys for hunting wild reindeer, and reindeer were also used for milking (Tegengren 1952; Aronsson 1991). Among this nomadic people, only some domestic reindeer performed various tasks, such as pulling sleds and carrying freight (e.g. castrated males; Korhonen 2008). In Siberia, reindeer herds could be very important, composed of several thousand individuals, as among Samoyedic (i.e. Nenets, Enets, Nganasans, Sepkups) or Ostyak (i.e. Khanty, Kets, Sepkups) peoples, while among Tungusic peoples (i.e. Evenks, Evens), reindeer is only used as a pack or saddle animal, although some northern groups occasionally use it both for riding and for drawing sledges (Mirov 1945; Willerslev et al. 2015). By contrast, in North America and Greenland, reindeer have never been domesticated by aboriginal people but the breeding of domestic individuals was introduced from Siberia and Norway in the 19th and 20th centuries, respectively (Klein 1980; Jepsen et al. 2002). Nowadays, in some parts of the world, reindeer are also used for racing or tourism (Salmi and Niinimäki 2016).

In any event, within these different cultures, there is a dichotomy between the groups that kept small herds under quite close supervision and high controlled mobility, in some cases keeping some captive individuals in corrals (e.g. Anderson et al. 2017); and others that let individuals roam freely. Prior to the transition to a reindeer-herding culture (before the 17th century), Sámis owned only small groups of 3–4 domestic individuals (Tegengren 1952). On the contrary, among the Komi, herds could be constituted of nearly 4,000 individuals, with the majority ranging freely and only a few dozen individuals kept near homes for domestic tasks (Dwyer and Istomin 2008). In addition, it has also been documented that some indigenous peoples frequently allowed local reindeer herds to crossbreed with wild individuals (Røed et al. 2008, 2014). For example, Evenk herders control the reproduction of their herds by occasionally allowing hybridization between wild males and domestic females (Anderson et al. 2017). Thus, given the complex current variability of breeding, the domestication process is even more difficult to understand since it has been gradual and does not appear to have been synchronous in the different regions nor with the same amplitude (Tegengren 1952; Lundmark 2007; Korhonen 2008; Bjørklund 2013). Despite the disparities observed in time and space, wild and domestic herds seem to have coexisted and still coexist very widely throughout Eurasia. It is mainly for these reasons that reindeer are considered to still be in the early phases of the domestication process (Baskin 2000; Reimers and Colman 2006). This provides a unique opportunity to analyse the interaction between domestic and wild forms, but could serve as an excellent model species to understand how the first domestication processes may have occurred.

However, this is all the more difficult to perceive since mainland northern Eurasia is home to two interbreeding reindeer subspecies ranging from Fennoscandia to northern Siberia: the mountain reindeer (*R.t. tarandus*), mainly inhabiting the Arctic tundra and the forest reindeer (*R.t. fennicus*), preferring the denser habitats of the taiga (Fig. 1b). More specifically, in Fennoscandia, domesticated reindeer were domesticated from wild mountain reindeer (Røed et al. 2008). Previously, the mountain reindeer was extant throughout the mountainous areas of northern Fennoscandia and forest reindeer were found throughout the taiga zone of northern Finland (Helle 1982). These two subspecies therefore seem to have had more marked biotopes in the past than today. However, perhaps in part due to the lack of a preliminary test on a large sample, the identification of these two subspecies from the bone elements is difficult. Previous studies have identified morphometric characters specific to each subspecies from cranial elements as well as body proportions of modern specimens (Nieminen and Helle 1980; Hakala et al. 1996). However, this remains difficult to apply to the fossil record, where the bones were often broken to obtain the marrow. Despite the importance of reindeer in the economy of past Arctic societies, archaeological remains for identifying subspecies are surprisingly rare (e.g. Puputti and Niskanen 2008, 2009). It is nevertheless possible to correctly discriminate the subspecies and sex of individuals from linear measurements on the postcranial skeleton, but this is more effective on complete bones or epiphyses (Puputti and Niskanen 2008, 2009). In this regard, Puputti and Niskanen (2009) indicate that the subspecies assessments from bones showing strong sexual dimorphism, and in particular the forelimb bones, are more prone to error; and therefore recommend to be prudent when assessing the subspecies of individuals of intermediate size.

Besides the use of destructive methods such as genetic analysis using ancient DNA (e.g. Røed et al. 2008; Bjørnstad et al. 2012; Salmi and Heino 2019) or geochemical analysis using stable isotopes (e.g. Britton et al. 2009; Salmi et al. 2015, 2020a) which can help distinguish wild versus domestic forms, traditional methodologies using measurements of and/or the bone morphology to identify them from archaeological deposits therefore suffer from different bias and none of these techniques allow for the robust identification of domestic individuals. In this study, we addressed these issues using 3D geometric morphometrics on a large set of long bones of the forelimb (i.e. humerus, radio-ulna and metacarpal) from a broad sample of modern Fennoscandian specimens and compared their discriminate potential. These bones are particularly important for understanding the differential feeding behaviours and activity patterns of domestic and wild reindeer. Indeed, it is historically known that during the winter, free-ranging reindeer dig for lichen buried beneath the snow using their forelimbs, while domestic animals were fed by owners when the crusting of the snow prevented animals from digging for food (Itkonen 1948; Helle 1982; Nieminen and Pietilä 1998; Korhonen 2008; Niinimäki and Salmi 2016). Indeed, free-ranging individuals can spend over eight hours per day and seven months per year doing this activity (Korhonen 2008). Among most Eurasian nomadic peoples, domestic reindeer may also be used to pull and carry loads, as well as for riding (Mirov 1945; Nieminen and Pietilä 1998; Inamura 2005; Dwyer and Istomin 2008; Korhonen 2008; Anderson et al. 2017). The analyses were performed on complete bones but the methodology has been adapted by focusing on the proximal and distal parts unaffected by entheseal changes and pathological lesions (Niinimäki and Salmi 2016; Salmi and Niinimäki 2016; Salmi et al. 2020b), as well as on the anatomical parts best preserved in the archaeological record in order to be complementary and directly applicable to fossil material (Owen et al. 2014; Cornette et al. 2015). The purpose of our study was to provide a reliable method of identifying domestic and wild individuals in modern reindeer populations from Fennoscandia, taking into account subspecies, sex and lifestyle, for an application to the archaeological contexts of the indigenous reindeer herders in Northern Eurasia. A better comprehension of the details of the domestication process and their implications on human-reindeer relationship could be the key to understanding the history of many present and past Arctic communities.

## Material and methods

### Modern reindeer sample

**Fig. 2:**
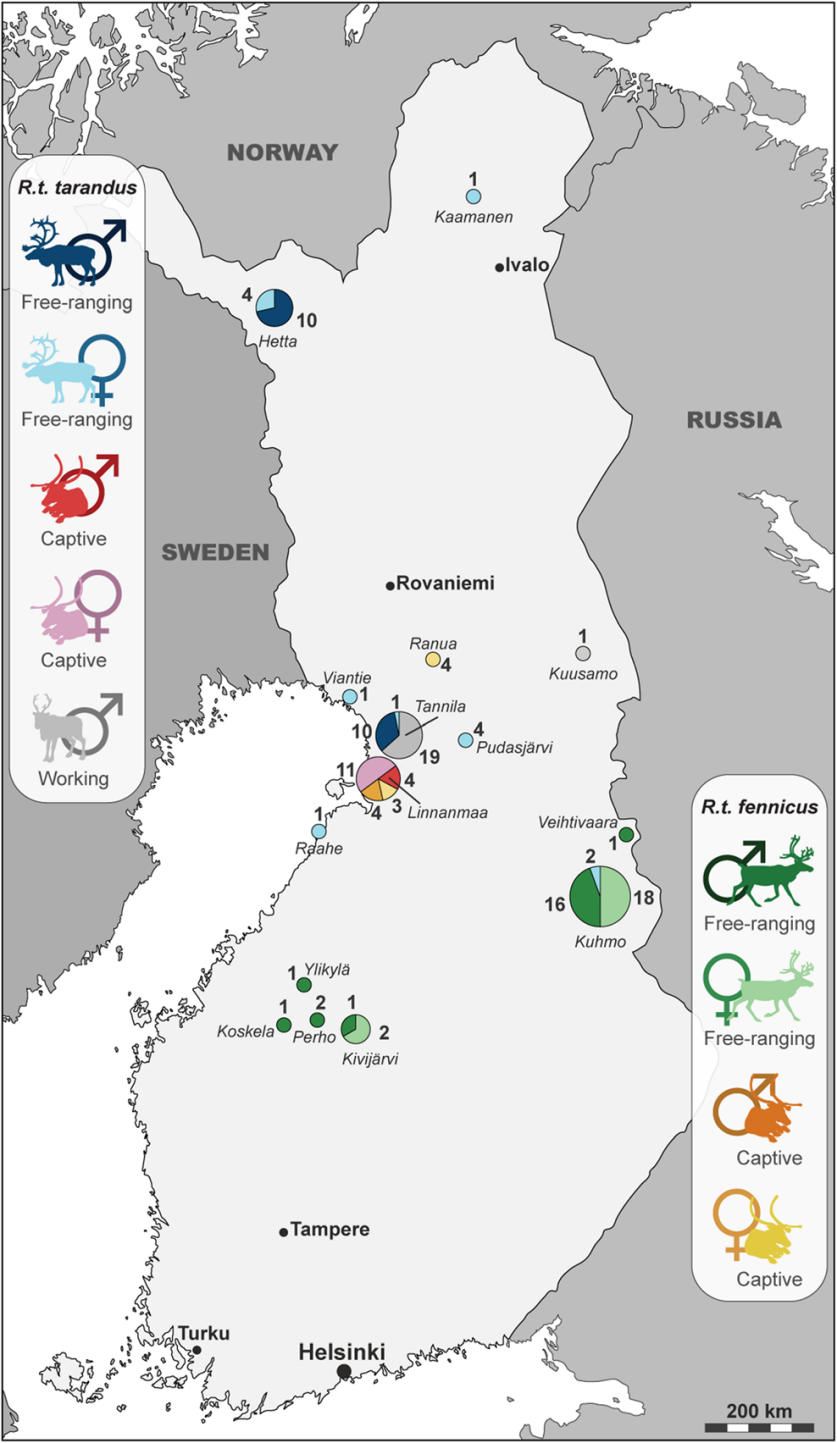
Location and details of effectives studied according to the subspecies (*R.t. tarandus* and *R.t. fennicus*), sex (male = ♂ and female = ♀) and lifestyle (free-ranging, captive and working)

In many mammals, the size and shape of skeletal and dental elements can be both influenced by intrinsic and extrinsic factors (Scott 1987; Kappelman 1988; DeGusta and Vrba 2005; Eronen et al. 2006; Klein et al. 2010; Polly 2010; Meachen et al. 2016; Ledevin et al. 2016; Renaud et al. 2018; Pelletier 2018, 2019; Souquet et al. 2019). It has been shown that some of these parameters, such as geographic position, topography or marked variations in climate and the environment, could also notably influence the body size and/or morphology of reindeer (Thomas and Everson 1981; Collins and Smith 1991; Weinstock 1997, 2002; Weladji and Holand 2003, 2006; Weladji et al. 2003; Reimers et al. 2005; Mårell et al. 2006; Magniez 2010). In order to limit these biases, the specimens analysed came mainly from central Finland (Fig. 2). The samples studied included the complete or partial skeletons of 123 individuals and represented the two subspecies currently present in Fennoscandia: the mountain reindeer (*R.t. tarandus*, *n* = 69) and the wild Finnish forest reindeer (*R.t. fennicus*, *n* = 54). All specimens were adults with fully fused epiphyses and sex was known (males, ♂ = 71 and females, ♀ = 52). Reindeer have probably been used for traction since the beginning of reindeer herding; while some were allowed to roam freely, others were kept in captivity. Working or captivity induce differential stress changes that can affect the shape and robusticity of bones. In this regard, Harbers et al. (2020) demonstrated in a recent study that the mobility reduction (i.e. captivity) induces a plastic response of the bone, leading to variations in the shape. Thus, lifestyle was also taken into account depending on whether the individuals were free-ranging (*n* = 77), captive (*n* = 26) or used for racing and pulling (*n* = 20). The details of effectives used for each bone are given in Table 1.

**Table 1:** Detail of specimens studied from Zoological Museum of Oulu according to the subspecies (*R.t. tarandus* and *R.t. fennicus*), sex (male = ♂ and female = ♀) and lifestyle (free-ranging, captive and working)

	Humerus		Radio-ulna		Metacarpal		Total
	*R. t. tarandus*	*R. t. fennicus*	*R. t. tarandus*	*R. t. fennicus*	*R. t. tarandus*	*R. t. fennicus*	
Free-ranging	7 ♂/8 ♀	22 ♂/20 ♀	7 ♂/9 ♀	23 ♂/20 ♀	20 ♂/14 ♀	22 ♂/20 ♀	43 ♂/34 ♀
Captivity	4 ♂/10 ♀	3 ♂/6 ♀	3 ♂/11 ♀	4 ♂/7 ♀	4 ♂/10 ♀	4 ♂/7 ♀	8 ♂/18 ♀
Working	9 ♂/0 ♀	0 ♂/0 ♀	9 ♂/0 ♀	0 ♂/0 ♀	15 ♂/0 ♀	0 ♂/0 ♀	20 ♂/0 ♀
Total	20 ♂/18 ♀	25 ♂/26 ♀	19 ♂/20 ♀	27 ♂/27 ♀	39 ♂/24 ♀	26 ♂/27 ♀	71 ♂/52 ♀

The material comprises zoo (University of Oulu and Ranua) and free-ranging reindeer from the collection of the Biodiversity Unit of the University of Oulu. In addition, some working and free-ranging male reindeer (*R.t. tarandus*) were collected by two of us (SN and AKS) during 2017–2019. This sample comprises 20 working male reindeer (three racing and one draught reindeer with stylopodial, zeugopodial and metapodial bones; five racing reindeer with stylopodial and zeugopodial bones; and 11 racing reindeer with metapodial bones). Most racing reindeer were from Tannila (Yli-Ii, Oulu Arc sub-region) and the draught reindeer was from Palosaari (Kuusamo, Koillismaa sub-region). The skeletons were prepared at the Biodiversity Unit at the University of Oulu and are currently archived at the Laboratory of Archaeology at the University of Oulu.

### Data acquisition

Geometric morphometrics (GMM) is a quantitative approach which allows the comparison of bone shapes and the visualization of significant morphological changes between groups of specimens while retaining the element of shape information related to size. In recent years, this methodology has been particularly developed to explore domestication and variability between populations, to study both the morphological variations of cranial and dental elements (Cucchi et al. 2011, 2017, 2019; Evin et al. 2013, 2015, 2017; Owen et al. 2014; Drake et al. 2015; Bopp-Ito et al. 2018; Duval et al. 2018) as well as those of postcranial elements (Bignon et al. 2005; Curran 2012; Barr 2014; Hanot et al. 2017, 2018; Haruda 2017; Haruda et al. 2019; Pöllath et al. 2019; Harbers et al. 2020). Thus, we applied a three-dimensional GMM approach to the computed tomography (CT) images of forelimb bones. CT scans were performed on a clinical CT (Somatom Definition Flash, Siemens Healthcare, Forcheim, Germany) using 120 kVp, 700 eff. mAs, 0.5-s rotation time, 0.6-mm slice thickness and increment, B70f reconstruction kernel, 140-mm reconstruction diameter, 0.35 pitch and 128 × 0.6 collimation. Each bone was scanned individually to avoid beam hardening artefacts.

**Fig. 3:**
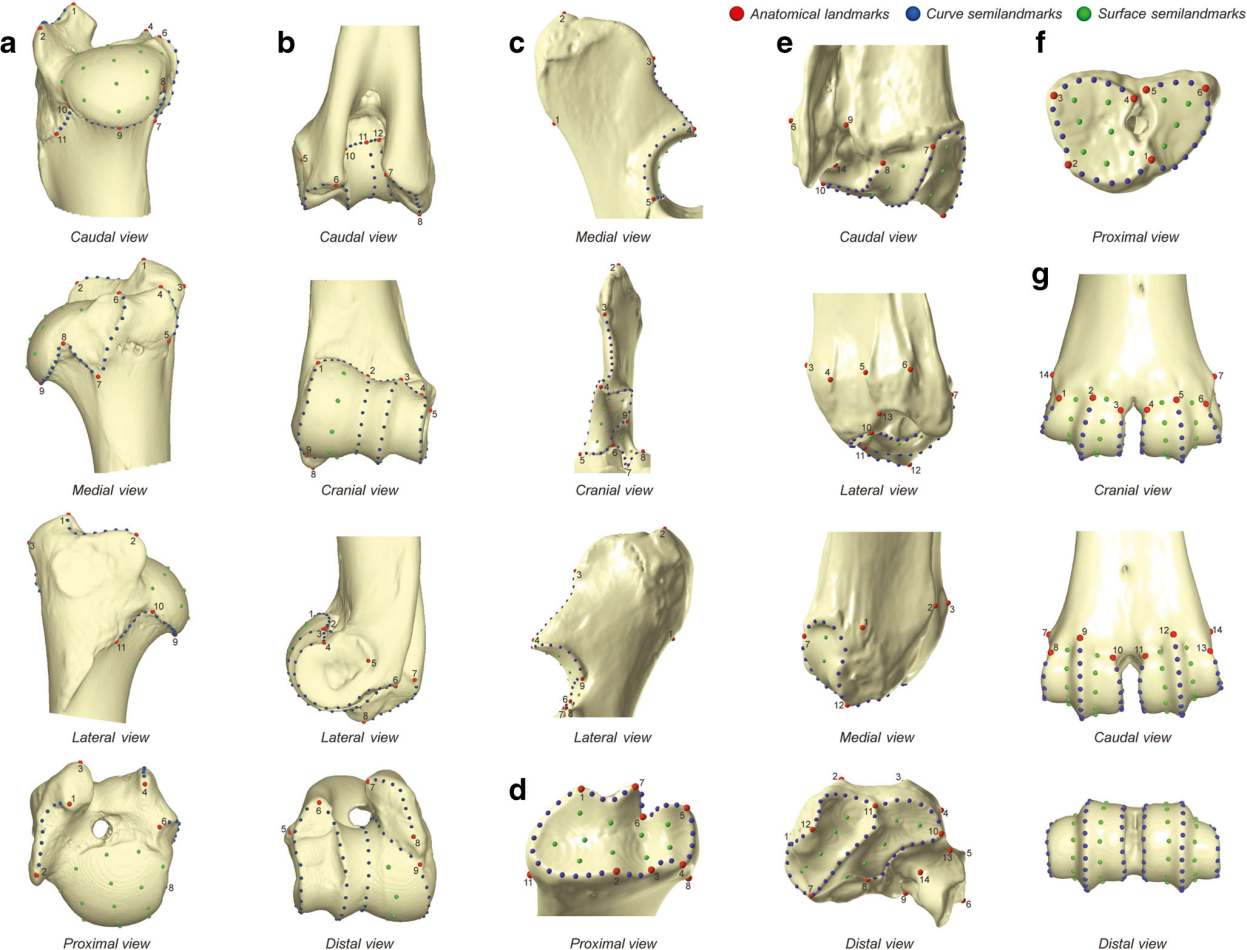
Landmarks and semilandmarks locations. (**a**) Proximal humerus; (**b**) distal humerus; (**c**) proximal ulna; (**d**) proximal radius; (**e**) distal radius; (**f**) proximal metacarpal; (**g**) distal metacarpal. The definitions of landmarks and semilandmarks are given in Table 2

For a better application to the fossil record, where bone extremities of the limbs are generally well-preserved, proximal and distal parts of each element were studied separately, focusing on the anatomical parts not affected by entheseal changes and pathological lesions (Niinimäki and Salmi 2016; Salmi and Niinimäki 2016; Salmi et al. 2020b). Due to the shape of articular surfaces, trochanters or condyles being difficult to quantify using traditional landmarks and the lack of homologous anatomical structures, semilandmarks have been included on curves and surfaces to help capture the three-dimensional structure of the epiphyses (Bookstein 1997). Thereby, 3D templates with 77 anatomical landmarks (ALMs), 339 curve semilandmarks (CSLM) and 92 surface semilandmarks (SSLM) have been digitized and warped to capture the form of the seven bony epiphyses (Fig. 3 and Table 2): proximal humerus (11 ALM, 7 curves, 49 CSLM, 14 SSLM), distal humerus (12 ALM, 11 curves, 89 CSLM, 5 SSLM), proximal ulna (9 ALM, 7 curves, 41 CSLM, 8 SSLM), proximal radius (11 ALM, 7 curves, 30 CSLM, 10 SSLM), distal radius (14 ALM, 5 curves, 42 CSLM, 10 SSLM), proximal metacarpal (6 ALM, 5 curves, 24 CSLM, 11 SSLM) and distal metacarpal (14 ALM, 6 curves, 64 CSLM, 34 SSLM). Digitization and warping were carried out using ViewBox v.4.0.1.7 software (dHAL software, Kifissia, Greece).

**Table 2:** Definition of anatomical landmarks and semilandmarks for each studied bone epiphyses shown in Fig. 3. *ALM*: anatomical landmarks; *CSLM*: curve semilandmarks; *SSLM*: surface semilandmarks

**Proximal humerus (a on Fig.** **3****)**			
**No.**	**ALM**	**No.**	**CSLM**
1	Upper tip of the greater tuberosity	12:19	Crest between ALM 1 and ALM 2 (n = 8)
2	Most antero-proximal point of the greater tuberosity crest	20:25	Crest between ALM 4 and ALM 5 (n = 6)
3	Most cranial point of the greater tuberosity crest	26:33	Crest between ALM 6 and ALM 7 (n = 8)
4	Upper tip of the lesser tuberosity	34:38	Crest between ALM 7 and ALM 8 (n = 5)
5	Tip of the convexity of the lesser tuberosity	39:46	Crest between ALM 8 and ALM 9 (n = 8)
6	Most medio-cranial point of the lesser tuberosity	47:54	Crest between ALM 9 and ALM 10 (n = 8)
7	Most disto-medial point of the lesser tuberosity	55:60	Crest between ALM 10 and ALM 11 (n = 6)
8	Most medio-caudal point of contact between the lesser tuberosity and the humeral head		
9	Disto-caudal tip of the humeral head	**No.**	**SSLM**
10	Latero-caudal point of contact between the greater tuberosity and the humeral head	61:74	Humeral head patch (n = 14)
11	Tip of the teres minor tuberosity		
**Distal humerus (b on Fig.** **3****)**			
**No.**	**ALM**	**No.**	**CSLM**
1	Most medio-proximal point of the cranial side of the trochlea	13:22	Crest between ALM 1 and ALM 9 (n = 10)
2	Maximum concavity of the cranial margin of the trochlea	23:37	Line between ALM 2 and ALM 11 (n = 15)
3	Latero-proximal point of contact between the trochlea and the capitulum of the cranial side	38:52	Crest between ALM 3 and ALM 6 (n = 15)
4	Most latero-proximal point of the cranial side of the capitulum	53:66	Crest between ALM 4 and ALM 6 (n = 14)
5	Point of the maximum of convexity of the lateral epicondyle crest	67:69	Crest between ALM 4 and ALM 3 (n = 3)
6	Most distal point of contact between the trochlea and the capitulum of the caudal side	70:73	Crest between ALM 3 and ALM 2 (n = 4)
7	Most postero-distal point of the medial epicondyle	74:80	Crest between ALM 2 and ALM 1 (n = 7)
8	Most antero-distal point of the medial epicondyle	81:88	Crest between ALM 7 and ALM 8 (n = 8)
9	Most disto-medial point of the cranial side of the trochlea	89:96	Line between ALM 9 and ALM 12 (n = 8)
10	Most latero-proximal point of the caudal side/margin of the trochlea	97:98	Crest between ALM 12 and ALM 11 (n = 2)
11	Maximum concavity of the caudal margin of the trochlea	99:101	Crest between ALM 11 and ALM 10 (n = 3)
12	Most medio-proximal point of the caudal side/margin of the trochlea	**No.**	**SSLM**
		102:106	Trochlea patch (n = 5)
**Proximal ulna (c on Fig.** **3****)**			
**No.**	**ALM**	**No.**	**CSLM**
1	Most postero-distal point of the olecranon process	10:19	Crest between ALM 3 and ALM 4 (n = 10)
2	Proximo-cranial tip of the olecranon process	20:27	Crest between ALM 4 and ALM 5 (n = 8)
3	Most anterior point of the olecranon process	28:31	Crest between ALM 5 and ALM 6 (n = 4)
4	Most anterior point of the proximal part of the trochlear notch	32:35	Crest between ALM 6 and ALM 9 (n = 4)
5	Most medial point of the trochlear notch	36:43	Crest between ALM 9 and ALM 4 (n = 8)
6	Most lateral point of contact between the trochlear notch and the radial notch	44:47	Crest between ALM 6 and ALM 7 (n = 4)
7	Most anterior point of the radial notch	48:50	Crest between ALM 7 and ALM 8 (n = 3)
8	Most lateral point of the radial notch	**No.**	**SSLM**
9	Most proximo-lateral point of the trochlear notch	51:58	Trochlear notch patch (n = 8)
**Proximal radius (d on Fig.** **3****)**			
**No.**	**ALM**	**No.**	**CSLM**
1	Most postero-medial point of the medial glenoid cavity	12:23	Crest between ALM 1 and ALM 2 (n = 12)
2	Most anterior point of the coronoid process	24:25	Crest between ALM 2 and ALM 3 (n = 2)
3	Point of maximum of concavity on the anterior part of the lateral glenoid cavity	26:27	Crest between ALM 3 and ALM 4 (n = 2)
4	Most antero-lateral point of the lateral glenoid cavity	28:31	Crest between ALM 4 and ALM 5 (n = 4)
5	Most postero-lateral point of the lateral glenoid cavity	32:35	Crest between ALM 5 and ALM 6 (n = 4)
6	Point of maximum of concavity on the posterior part of the lateral glenoid cavity	36:37	Crest between ALM 6 and ALM 7 (n = 2)
7	Point of maximum of convexity on the posterior part of the lateral glenoid cavity	38:41	Crest between ALM 7 and ALM 1 (n = 4)
8	Most antero-distal point of the lateral tuberosity		
9	Most postero-distal point of the lateral tuberosity	**No.**	**SSLM**
10	Most postero-distal point of the medial tuberosity	42:51	Radial head surface patch (n = 10)
11	Most antero-distal point of the medial tuberosity		
**Distal radius (e on Fig.** **3****)**			
**No.**	**ALM**	**No.**	**CSLM**
1	Most medial point of the transverse ridge of distal radius	15:20	Crest between ALM 10 and ALM 11 (n = 6)
2	Most proximo-medial point of the groove for the radial carpal extensor	21:28	Crest between ALM 11 and ALM 12 (n = 8)
3	Most proximo-lateral point of the groove for the radial carpal extensor	29:38	Crest between ALM 12 and ALM 7 (n = 10)
4	Most proximo-medial point of the groove for finger extensors	39:48	Crest between ALM 7 and ALM 11 (n = 10)
5	Most proximo-lateral point of the groove for finger extensors	49:56	Crest between ALM 8 and ALM 10 (n = 8)
6	Most lateral point of the transverse ridge of distal radius		
7	Most postero-lateral point of the medial facet of distal radius		
8	Most postero-lateral point of the intermediate facet of distal radius	**No.**	**SSLM**
9	Point of maximum of concavity of the sulcus of the transverse ridge of distal radius	57:61	Medial articular surface patch (n = 5)
10	Most lateral point of the intermediate facet of distal radius	62:66	Lateral articular surface patch (n = 5)
11	Most antero-lateral point of the medial facet of distal radius		
12	Most medial point of the medial facet of distal radius		
13	Most lateral point of the lateral facet of distal radius		
14	Most postero-medial point of the lateral facet of distal radius		
**Proximal metacarpal (f on Fig.** **3****)**			
**No.**	**ALM**	**No.**	**CSLM**
1	Most anterior point of contact between the articular surface for the hamate bone and the	7:11	Crest between ALM 1 and ALM 2 (n = 5)
	articular surface for the capitato-trapezoid bone	12:15	Crest between ALM 2 and ALM 3 (n = 4)
2	Most antero-medial point of the articular surface for the capitato-trapezoid bone	16:20	Crest between ALM 3 and ALM 4 (n = 5)
3	Most postero-medial point of the articular surface for the capitato-trapezoid bone	21:23	Crest between ALM 5 and ALM 6 (n = 3)
4	Most postero-lateral point of the articular surface for the capitato-trapezoid bone	24:30	Crest between ALM 6 and ALM 1 (n = 7)
5	Most postero-medial point of the articular surface for the hamate bone	**No.**	**SSLM**
6	Most postero-lateral point of the articular surface for the hamate bone	31:41	Proximal articular surface patch (n = 11)
**Distal metacarpal (g on Fig.** **3****)**			
**No.**	**ALM**	**No.**	**CSLM**
1	Most medio-proximal point of the anterior outline of the medial articular eminence (MAE)	15:22	Crest between ALM 1 and ALM 13 (n = 8)
2	Most proximal point of the anterior outline of the medial articular eminence	23:36	Crest between ALM 2 and ALM 12 (n = 14)
3	Most latero-proximal point of the anterior outline of the medial articular eminence	37:46	Crest between ALM 3 and ALM 11 (n = 10)
4	Most medio-proximal point of the anterior outline of the lateral articular eminence (LAE)	47:56	Crest between ALM 4 and ALM 10 (n = 10)
5	Most proximal point of the anterior outline of the lateral articular eminence	57:70	Crest between ALM 5 and ALM 9 (n = 14)
6	Most latero-proximal point of the anterior outline of the lateral articular eminence	71:78	Crest between ALM 6 and ALM 8 (n = 8)
7	Most latero-proximal point of the posterior outline of the lateral articular eminence		
8	Most proximal point of the posterior outline of the lateral articular eminence		
9	Most medio-proximal point of the posterior outline of the lateral articular eminence	**No.**	**SSLM**
10	Most latero-proximal point of the posterior outline of the medial articular eminence	79:86	Medial trochlear patch on MAE (n = 8)
11	Most proximal point of the posterior outline of the medial articular eminence	87:95	Lateral trochlear patch on MAE (n = 9)
12	Most medio-proximal point of the posterior outline of the medial articular eminence	96:104	Medial trochlear patch on LAE (n = 9)
13	Most proximo-medial point of the ligament insertion fossa	05:112	Lateral trochlear patch on LAE (n = 8)
14	Most proximo-lateral point of the ligament insertion fossa		

### Size and shape analyses

Unlike landmarks, semilandmarks do not have an exact anatomical correspondence on the structure of the epiphyses, and instead they were allowed to slide along curves and surfaces in order to minimize the bending energy of the thin-plate spline (TPS) interpolation function (Bookstein 1997; Gunz et al. 2005; Gunz and Mitteroecker 2013). After sliding, all specimen coordinates were aligned using the Generalized Procrustes Analysis (GPA, Rohlf and Slice 1990; Bookstein 1991, 1996). All configurations were translated and rotated to minimize the overall sum of the squared distances between the corresponding landmarks and semilandmarks. To remove the effects of scale, GPA also computes a unit centroid size as the square root of the summed squared distances from all landmarks and semilandmarks to their centroid (Bookstein 1996; Dryden and Mardia 1998).

Size differences were evaluated from log-transformed centroid sizes for the seven epiphyses analysed by pooling the specimens by (1) subspecies, (2) sexes, (3) lifestyles, (4) ‘subspecies + sexes’ and (5) ‘subspecies + sexes + lifestyles’, using Kruskal-Wallis tests with an error threshold set at α = 5%. Pairwise comparisons of the populations were performed using multiple Wilcoxon rank tests according to these different categories. To control for the false discovery rate, a multicomparison correction was applied to *P* values using the ‘Benjamini-Hochberg’ method (Benjamini and Hochberg 1995). Shape differences between these different groups were estimated using a multivariate analysis of variance (MANOVA), with significant interaction (α = 5%) assumed to reflect population differences. Shape variation was visualized using a principal component analysis (PCA) based on Procrustes coordinates. To better apprehend variations along the principal axes, we created a 3D digital mesh for each of the elements that were warped toward the Procrustes grand mean using a thin-plate spline (TPS) interpolation function (Bookstein 1991). The visualizations of shapes at the extremes of the principal component axes were performed from the surface of the Procrustes mean configuration (Wiley et al. 2005), with magnification by a scale factor of 0.1. We then assessed the assignment accuracy for the seven bony elements by calculating the cross-validated correct classification percentages on the reference sample for each category, using a canonical analysis of variance (CVA). In order not to affect the results of cross-validation, we reduced the dimensionality of our data set by keeping the values of the main components expressing 95% of the total variance before each canonical analysis (Kovarovic et al. 2011). Finally, allometry was assessed using multivariate regressions of shape variables on the log-transformed centroid sizes. All morphometric statistics were performed with Rstudio v.1.1.383 (R Development Core Team 2011), using ‘MASS’ (Venables and Ripley 2002), ‘ade4’ (Dray and Dufour 2007) and ‘geomorph’ (Adams and Otárola-Castillo 2013) libraries.

## Results

### Size variation of skeletal elements

The results of the Kruskal-Wallis tests on size data reveal significant differences between all categories, namely subspecies, sexes, lifestyles and ‘subspecies + sexes’, as well as ‘subspecies + sexes + lifestyles’, for all bony elements (*P* < < 0.01; Table 3). All seven analysed epiphyses of the forelimb long bones displayed the same pattern of size differentiation among subspecies, sexes and lifestyles. In general, pairwise comparisons revealed that the forest reindeer (*R.t. fennicus*) is significantly bigger than the mountain reindeer (*R.t. tarandus*), as well as males are significantly bigger than females (all *P* < < 0.01). Male *R.t. fennicus*, female *R.t. fennicus*, male *R.t. tarandus* and female *R.t. tarandus* all showed significant differences between them for all bony elements (all *P* < < 0.01), except specifically between female *R.t. fennicus* and male *R.t. tarandus* for the proximal and distal parts of the metacarpals (*P* = 0.727 and *P* = 0.678, respectively). Thereby, male *R.t. fennicus* have the largest bony elements, female *R.t. tarandus* are the smallest and finally male *R.t. tarandus* are slightly bigger than female *R.t. fennicus*. Regarding lifestyles, free-ranging and working individuals were not statistically different (all *P* > 0.05). However, captive individuals were significantly smaller than free-ranging and working individuals (all *P* < < 0.01).

**Table 3:** Results of the Kruskal-Wallis tests for each element analysed according to the different categories. A significant contribution was considered for *P* value < 0.05 (in italics)

	Df	Chi-square	*P* value
Proximal humerus			
Subspecies (ssp.)	1	12.079	*5.101e−04*
Sex	1	48.539	*3.238e−12*
Lifestyle	2	16.586	*2.502e−04*
ssp. × sex	3	62.432	*1.776e−13*
ssp. × sex × lifestyle	8	68.654	*9.105e−12*
Distal humerus			
Subspecies (ssp.)	1	12.780	*3.503e−04*
Sex	1	42.829	*5.974e−11*
Lifestyle	2	19.649	*5.410e−05*
ssp. × sex	3	57.573	*1.939e−12*
ssp. × sex × lifestyle	8	64.655	*5.646e−11*
Proximal radius			
Subspecies (ssp.)	1	14.495	*1.405e−04*
Sex	1	42.361	*7.589e−11*
Lifestyle	2	20.263	*3.981e−05*
ssp. × sex	3	56.724	*2.943e−12*
ssp. × sex × lifestyle	8	65.747	*3.435e−11*
Distal radius			
Subspecies (ssp.)	1	12.669	*3.717e−04*
Sex	1	49.692	*1.799e−12*
Lifestyle	2	21.947	*1.715e−05*
ssp. × sex	3	62.537	*1.686E−13*
ssp. × sex × lifestyle	8	72.474	*1.579e−12*
Proximal ulna			
Subspecies (ssp.)	1	12.883	*3.317e−04*
Sex	1	54.969	*1.225e−13*
Lifestyle	2	20.543	*3.461e−05*
ssp. × sex	3	67.575	*1.411e−14*
ssp. × sex × lifestyle	8	76.028	*3.070e−13*
Proximal metacarpal			
Subspecies (ssp.)	1	28.516	*9.295e−08*
Sex	1	34.731	*3.786e−09*
Lifestyle	2	17.163	*1.876e−04*
ssp. × sex	3	72.765	*1.092e−15*
ssp. × sex × lifestyle	8	87.480	*1.509e−15*
Distal metacarpal			
Subspecies (ssp.)	1	29.954	*4.424e−08*
Sex	1	36.054	*1.919e−09*
Lifestyle	2	16.489	*2.628e−04*
ssp. × sex	3	75.935	*2.284e−16*
ssp. × sex × lifestyle	8	88.220	*1.068e−15*

**Fig. 4:**
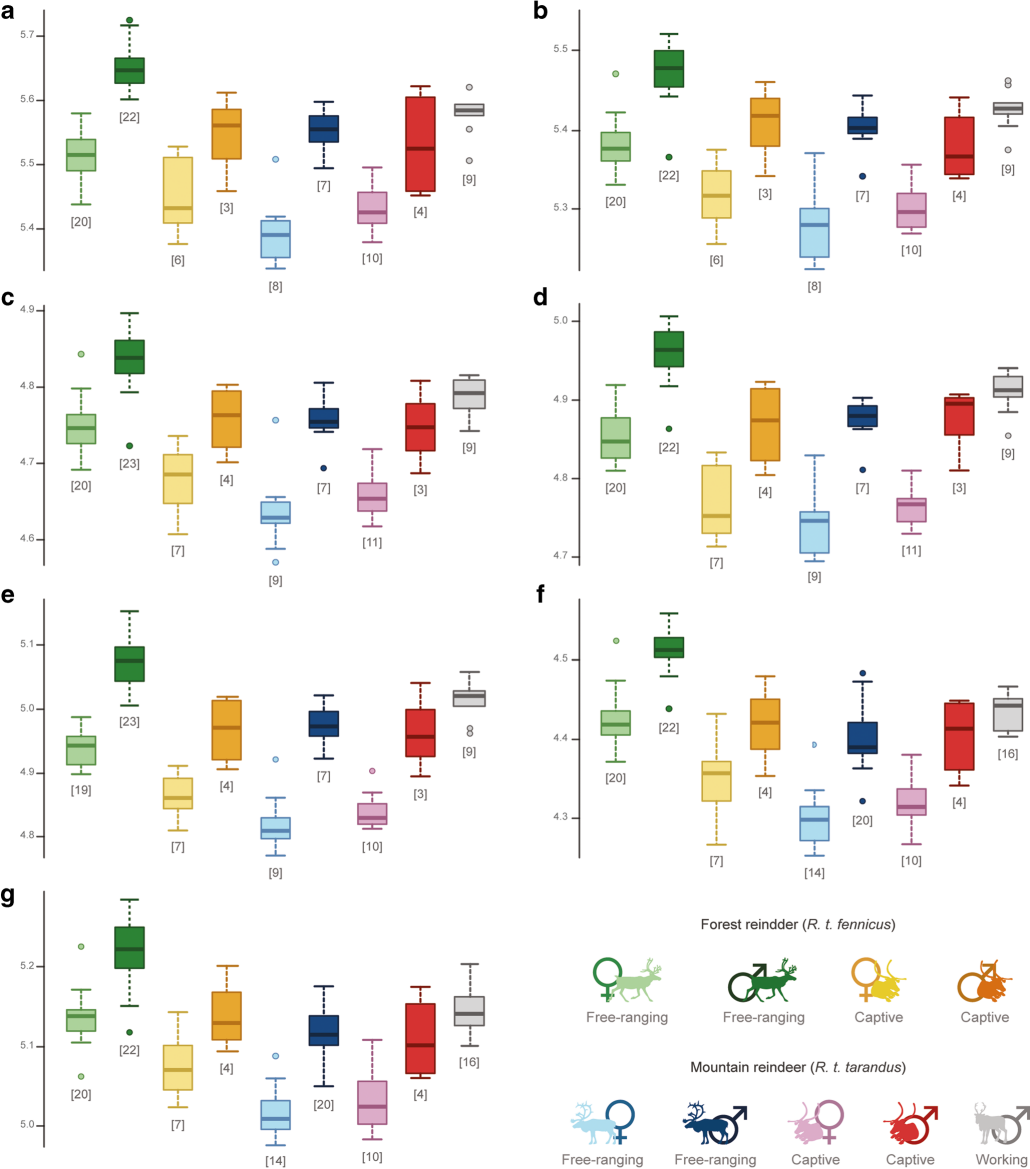
Boxplots of the variation in log-transformed centroid size according to the subspecies (*R.t. tarandus* and *R.t. fennicus*), sex (male = ♂ and female = ♀) and lifestyle (free-ranging, captive and working). (**a**) Proximal humerus; (**b**) distal humerus; (**c**) proximal radius; (**d**) distal radius; (**e**) proximal ulna; (**f**) proximal metacarpal; (**g**) distal metacarpal. The numbers in square brackets represent the number of bones analysed by elements in each group

Specificities were observed when the specimens were divided into nine groups, comprising at the same time the subspecies, sex and lifestyle (Fig. 4 and Table 4). Except for the distal epiphysis of the humerus (*P* = 0.095), significant differences have always been found between free-ranging male *R.t. fennicus* and all other groups (all *P* < 0.05), corresponding to a bigger bone size. Contrariwise, free-ranging female *R.t. tarandus* corresponded to a smaller bone size and were significantly different than of all other populations (all *P* < 0.05), except captive females in both subspecies (all *P* > 0.05). In *R.t. fennicus*, captive male and female individuals were significantly smaller than their wild counterparts (i.e. free-ranging). In contrast, in *R.t. tarandus*, this size difference was not significant in males and females between captive and free-ranging individuals, respectively. Furthermore, in most cases, there were important overlaps in the size range for all bony elements. No significant differences were found between free-ranging female *R.t. fennicus*, captive male *R.t. fennicus*, free-ranging male *R.t. tarandus*, captive male *R.t. tarandus* and working male *R.t. tarandus*, although free-ranging female *R.t. fennicus* seem to have smaller bony elements and the working male *R.t. tarandus* have larger bones.

**Table 4:** Multi-test comparisons (*P* values) of log-transformed centroid sizes for each element analysed between the different reindeer groups (pairwise Wilcoxon rank tests after the ‘Benjamini-Hochberg’ correction). A significant contribution was considered for *P* value < 0.05 (in italics)

	*R.t. fennicus* ♀ free-ranging	*R.t. fennicus* ♀ captive	*R.t. fennicus* ♂ free-ranging	*R.t. fennicus* ♂ captive	*R.t. tarandus* ♀ free-ranging	*R.t. tarandus* ♀ captive	*R.t. tarandus* ♂ free-ranging	*R.t. tarandus* ♂ working
Proximal humerus								
*R.t. fennicus* ♀ captive	*0.041*	*–*	–	–	–	–	–	–
*R.t. fennicus* ♂ free-ranging	*1.698e−07*	*1.299e−04*	–	–	–	–	–	–
*R.t. fennicus* ♂ captive	0.502	0.132	*0.0137*	*–*	–	–	–	–
*R.t. tarandus* ♀ free-ranging	*2.471e−04*	0.064	*8.201e−06*	*0.042*	–	–	–	–
*R.t. tarandus* ♀ captive	*8.328e−05*	0.892	*1.116e−06*	*0.046*	0.120	–	–	–
*R.t. tarandus* ♂ free-ranging	*0.022*	*0.017*	*2.471e−04*	1.000	*0.001*	*3.364e−04*	–	–
*R.t. tarandus* ♂ working	*3.364e−04*	*0.004*	*1.352e−04*	0.720	*4.935e−04*	*1.299e−04*	0.147	–
*R.t. tarandus* ♂ captive	0.982	0.147	*0.005*	1.000	*0.031*	*0.050*	0.982	0.825
Distal humerus								
*R.t. fennicus* ♀ captive	*0.006*	*–*	–	–	–	–	–	–
*R.t. fennicus* ♂ free-ranging	*6.624e−05*	*2.230e−04*	–	–	–	–	–	–
*R.t. fennicus* ♂ captive	0.515	0.143	0.095	–	–	–	–	–
*R.t. tarandus* ♀ free-ranging	*3.938e−04*	0.189	*1.640e−05*	*0.042*	–	–	–	–
*R.t. tarandus* ♀ captive	*1.438e−05*	0.497	4.464e**−**06	*0.027*	0.294	–	–	–
*R.t. tarandus* ♂ free-ranging	0.149	*0.010*	*0.002*	0.564	*0.002*	*8.228e−04*	–	–
*R.t. tarandus* ♂ working	*0.002*	*0.001*	*0.004*	0.635	*3.938e−04*	*1.559e−04*	0.111	–
*R.t. tarandus* ♂ captive	0.737	0.158	*0.008*	0.647	*0.029*	*0.016*	0.495	0.191
Proximal radius								
*R.t. fennicus* ♀ captive	*0.002*	–	–	–	–	–	–	–
*R.t. fennicus* ♂ free-ranging	*1.807e−05*	*2.653e−04*	–	–	–	–	–	–
*R.t. fennicus* ♂ captive	0.767	*0.040*	*0.012*	–	–	–	–	–
*R.t. tarandus* ♀ free-ranging	*4.332e−04*	0.126	*1.027e−05*	*0.021*	–	–	–	–
*R.t. tarandus* ♀ captive	*3.775e−06*	0.394	*3.775e−06*	*0.007*	0.116	–	–	–
*R.t. tarandus* ♂ free-ranging	0.464	*0.009*	*0.002*	1.000	*0.005*	*5.028e−04*	–	–
*R.t. tarandus* ♂ working	*0.008*	*6.293e−04*	*0.003*	0.255	*4.231e−04*	*8.575e−05*	0.063	–
*R.t. tarandus* ♂ captive	1.000	0.156	*0.040*	1.000	*0.050*	*0.040*	0.909	0.350
Distal radius								
*R.t. fennicus* ♀ captive	*0.002*	–	–	–	–	–	–	–
*R.t. fennicus* ♂ free-ranging	*6.839e−07*	*3.595e−05*	–	–	–	–	–	–
*R.t. fennicus* ♂ captive	0.647	0.064	*0.010*	–	–	–	–	–
*R.t. tarandus* ♀ free-ranging	*3.595e−05*	0.421	*1.786e−06*	*0.010*	–	–	–	–
*R.t. tarandus* ♀ captive	*4.252e−07*	0.930	*3.719e−07*	*0.006*	0.174	–	–	–
*R.t. tarandus* ♂ free-ranging	0.223	*0.005*	*6.957e−04*	0.930	*9.684e−04*	*2.514e−04*	–	–
*R.t. tarandus* ♂ working	*2.581e−04*	*5.721e−04*	*0.002*	0.191	*1.851e−04*	*6.125e−05*	*0.027*	–
*R.t. tarandus* ♂ captive	0.647	0.162	*0.016*	0.908	*0.028*	*0.010*	0.727	0.144
Proximal ulna								
*R.t. fennicus* ♀ captive	*5.558e−04*	–	–	–	–	–	–	–
*R.t. fennicus* ♂ free-ranging	*7.250e−09*	*8.626e−06*	–	–	–	–	–	–
*R.t. fennicus* ♂ captive	0.419	*0.038*	*0.003*	–	–	–	–	–
*R.t. tarandus* ♀ free-ranging	*5.212e−05*	0.058	*8.556e−07*	*0.011*	–	–	–	–
*R.t. tarandus* ♀ captive	*8.626e−06*	0.135	*3.890e−07*	*0.004*	0.188	–	–	–
*R.t. tarandus* ♂ free-ranging	*0.038*	*0.001*	*1.592e−04*	0.954	*8.992e−04*	*3.364e−04*	–	–
*R.t. tarandus* ♂ working	*9.742e−05*	*5.244e−04*	*9.554e−04*	0.135	*1.592e−04*	*9.742e−05*	*0.045*	–
*R.t. tarandus* ♂ captive	0.783	0.089	*0.038*	1.000	*0.031e−02*	*0.025*	0.882	0.419
Proximal metacarpal								
*R.t. fennicus* ♀ captive	*0.003*	–	–	–	–	–	–	–
*R.t. fennicus* ♂ free-ranging	*2.164e−07*	*8.388e−06*	–	–	–	–	–	–
*R.t. fennicus* ♂ captive	0.970	0.296	*0.002*	–	–	–	–	–
*R.t. tarandus* ♀ free-ranging	*3.449e−08*	0.100	*9.740e−09*	*0.003*	–	–	–	–
*R.t. tarandus* ♀ captive	*6.845e−07*	0.278	*2.164e−07*	*0.007*	0.294	–	–	–
*R.t. tarandus* ♂ free-ranging	*0.011*	*0.012*	*9.740e−09*	0.554	*2.144e−06*	*8.988e−06*	–	–
*R.t. tarandus* ♂ working	0.322	*0.001*	*1.162e−06*	0.725	*2.164e−07*	*2.203e−06*	*0.004*	–
*R.t. tarandus* ♂ captive	0.708	0.296	*3.399e−03*	0.937	*0.005*	*0.007*	0.937	0.322
Distal metacarpal								
*R.t. fennicus* ♀ captive	*0.006*	–	–	–	–	–	–	–
*R.t. fennicus* ♂ free-ranging	*2.372e−08*	*4.192e−06*	–	–	–	–	–	–
*R.t. fennicus* ♂ captive	0.701	0.101	*0.009*	–	–	–	–	–
*R.t. tarandus* ♀ free-ranging	*2.587e−08*	*0.008*	*9.482e−09*	*0.002*	–	–	–	–
*R.t. tarandus* ♀ captive	*1.864e−06*	0.083	*1.594e−07*	*0.008*	0.524	–	–	–
*R.t. tarandus* ♂ free-ranging	0.097	*0.026*	*5.605e−10*	0.524	*1.241e−07*	*1.338e−05*	–	–
*R.t. tarandus* ♂ working	0.546	*0.003*	*1.162e−06*	0.562	*1.547e−07*	*4.192e−06*	*0.012*	–
*R.t. tarandus* ♂ captive	0.499	0.562	*7.410e−04*	0.546	*0.006*	*0.038*	0.794	0.409

### Shape variation of skeletal elements

Except for proximal epiphyses of the radius and metacarpal, MANOVA analyses revealed at least a significant difference in shape among some groups for all the other elements, although it varies according to the bone or the category investigated (Table 5). In particular, shapes of the distal humerus and radius were significantly different between subspecies, while lifestyle is rather perceptible through the shapes of the humerus (proximal and distal) and the distal epiphysis of the radius. However, unlike sizes of the bone elements (see above), shape does not differentiate sex only (except for the distal radius). Thus, no significant difference was noted for the ‘subspecies + sex’ category (with the exception of the ulna proximal). On the other hand, significant differences were found for the proximal parts of the humerus and ulna, and the distal part of the metacarpals among the ‘subspecies + sexes + lifestyles’ category. Cross-validated classification rates vary quite widely depending on the groups and bones (Table 5). The best classification results obtained (> 85%) generally concerned the subspecies and sex, and were more effective for the humerus (proximal and distal) and the distal element of the metacarpal. Lifestyle alone was generally ranked between 70 and 85%. However, ‘subspecies + sex’ and ‘subspecies + sexes + lifestyles’ subgroups had the least effective classification results (< 70%).

**Table 5:** Results of the MANOVA tests for each element analysed and the percentage of correct cross-validated classification (CCV) according to the different categories. A significant contribution was considered for *P* value < 0.05 (in italics)

	Df	Pillai	Approx F	Num Df	Den Df	Pr	CCV (%)
Proximal humerus							
Subspecies (ssp.)	1	0.998	5.223	87	1	0.337	82.02
Sex	1	0.999	41.960	87	1	0.122	92.13
Lifestyle	2	1.992	5.770	172	4	*0.048*	86.52
ssp. × sex	3	2.951	2.119	255	9	0.107	73.03
ssp. × sex × lifestyle	8	7.527	1.591	640	64	*0.011*	58.43
Distal humerus							
Subspecies (ssp.)	1	0.999	331.390	86	2	*0.003*	86.52
Sex	1	0.978	0.500	87	1	0.839	83.15
Lifestyle	2	1.987	5.594	170	6	*0.018*	80.90
ssp. × sex	3	2.946	1.923	255	9	0.140	66.29
ssp. × sex × lifestyle	8	7.553	1.689	640	64	*0.005*	58.43
Proximal radius							
Subspecies (ssp.)	1	0.994	1.830	91	1	0.538	78.49
Sex	1	0.991	1.243	91	1	0.628	73.19
Lifestyle	2	1.988	3.687	180	4	0.104	72.04
ssp. × sex	3	2.943	1.734	267	9	0.184	62.37
ssp. × sex × lifestyle	8	7.378	1.129	672	64	0.276	43.01
Distal radius							
Subspecies (ssp.)	1	0.999	301.420	90	1	*0.046*	70.65
Sex	1	0.999	1875.500	90	1	*0.018*	90.22
Lifestyle	2	1.994	7.801	178	4	*0.028*	78.26
ssp. × sex	3	2.960	2.550	264	9	0.062	66.30
ssp. × sex × lifestyle	8	7.414	1.219	664	64	0.161	53.26
Proximal ulna							
Subspecies (ssp.)	1	0.990	1.082	89	1	0.661	71.43
Sex	1	0.997	3.871	89	1	0.388	89.01
Lifestyle	2	1.971	1.527	176	4	0.377	76.92
ssp. × sex	3	2.970	3.474	261	9	*0.022*	65.93
ssp. × sex × lifestyle	8	7.691	2.427	656	64	*1.349e−05*	51.65
Proximal metacarpal							
Subspecies (ssp.)	1	0.999	32.627	114	1	0.139	81.90
Sex	1	0.999	15.937	114	1	0.197	71.55
Lifestyle	2	1.967	1.040	226	4	0.571	67.24
ssp. × sex	3	2.947	1.479	336	9	0.269	50.86
ssp. × sex × lifestyle	8	7.486	1.088	856	64	0.344	38.79
Distal metacarpal							
Subspecies (ssp.)	1	0.999	15.990	114	1	0.197	82.76
Sex	1	0.995	1.758	114	1	0.548	88.79
Lifestyle	2	1.976	1.483	226	4	0.390	70.69
ssp. × sex	3	2.864	0.566	336	9	0.927	65.52
ssp. × sex × lifestyle	8	7.599	1.418	856	64	*0.039*	48.28

**Fig. 5:**
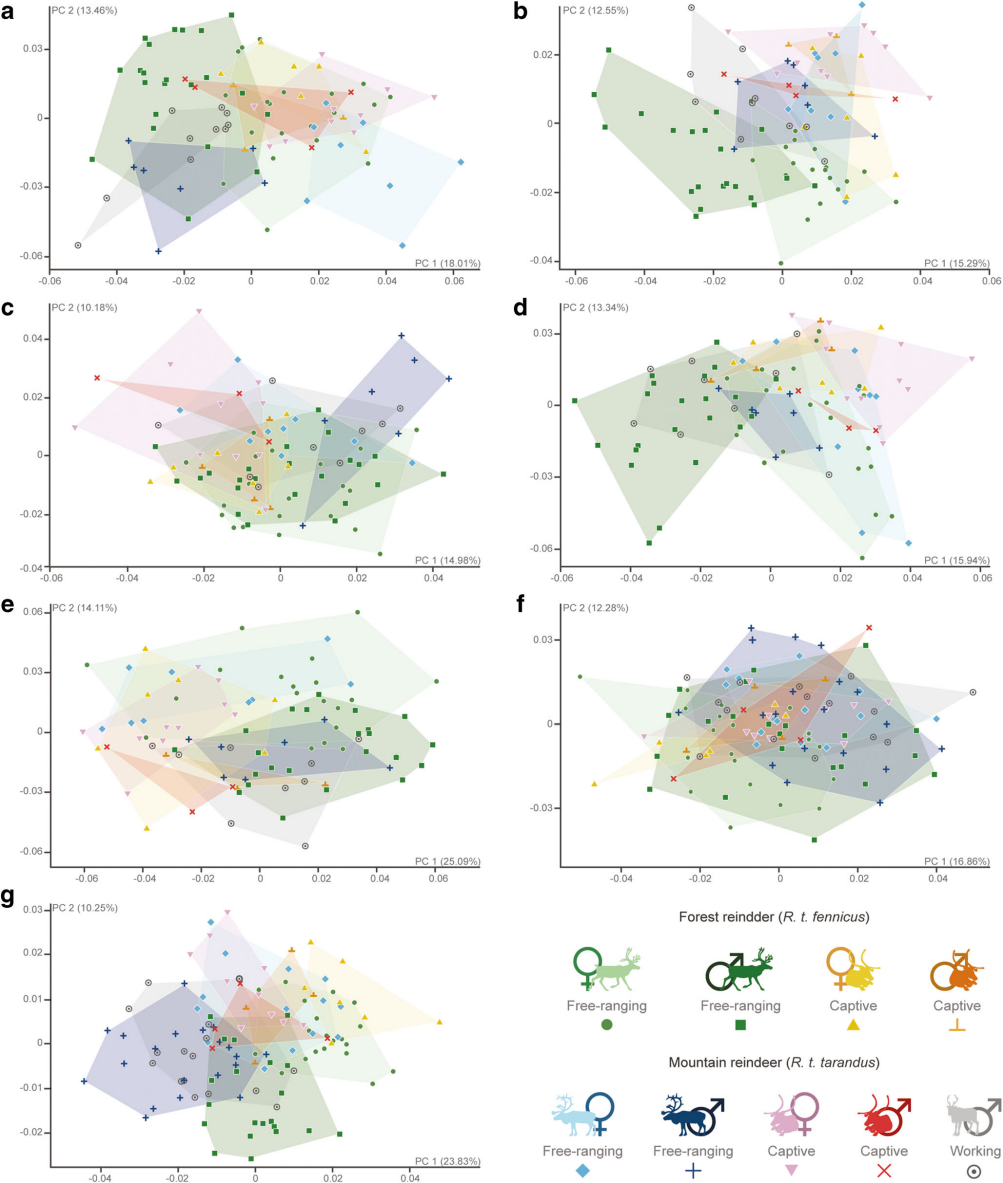
Scatter plots of the two first axes (PC 1 and PC 2) of principal component analyses performed on the shape data according to the subspecies (*R.t. tarandus* and *R.t. fennicus*), sex (male = ♂ and female = ♀) and lifestyle (free-ranging, captive and working). (**a**) Proximal humerus; (**b**) distal humerus; (**c**) proximal radius; (**d**) distal radius; (**e**) proximal ulna; (**f**) proximal metacarpal; (**g**) distal metacarpal. The proportion of the total variance respectively expressed by the axes PC1 and PC2 is indicated in brackets

**Fig. 6:**
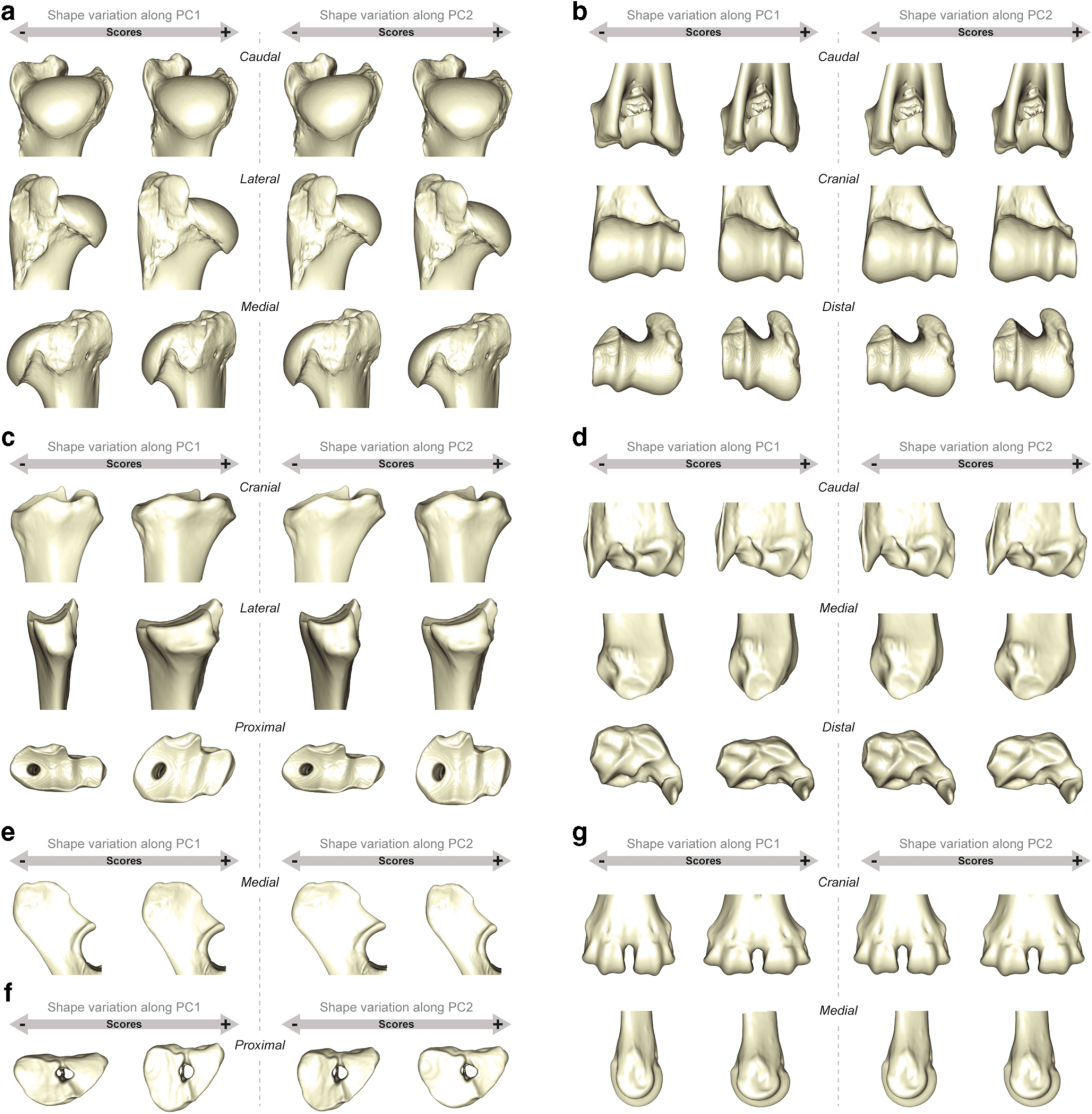
Visualization of shape variation via deformation of the mean shape along negative and positive PC1 and PC2 values (magnified by a scale factor of 0.1). **a** Proximal humerus; **b** distal humerus; **c** proximal radius; **d** distal radius; **e** proximal ulna; **f** proximal metacarpal; **g** distal metacarpal

Despite the overlaps, the patterns of variation seem to evolve in the same way in each bone element (Fig. 5). Free-ranging females and males for both subspecies showed similar variations in morphospace: shape variation along the first principal component (PC1) revealed sexual variation (accounting for between 15 and 25% of the shape variation according to the bony elements), in particular for the humerus, radius and the distal part of the metacarpal. However, along the second principal component (PC2), shape variation was expressed more through lifestyle, i.e. free-ranging versus captive individuals (accounting for between 10 and 15% of the shape variation). On the contrary, for the ulna, PC1 expressed the shape variation for the lifestyle (25.09% of the total variance) and PC2 for the sex (14.11% of the total variance). Only the proximal epiphysis of the metacarpal did not seem to show preferential variations according to the different groups. Working individuals (i.e. male *R.t. tarandus*) did not differ from their free-ranging counterparts. However, despite a reduced sample size for captive individuals, they still seemed to present morphological variations compared to free-ranging individuals. For each bone, the theoretical shape variations along the PC1 and PC2 generally corresponded to a more robust morphology in males and a more slender morphology in females, just as captive individuals trended towards greater gracility than free-ranging individuals (Figs. 5 and 6):

- Humerus: For the proximal humerus epiphysis, morphological variation along the negative values of the PC1 showed a massive morphology, with mediolaterally and craniocaudally broad epiphyses and a wide humeral head. These are features that were more associated with male criteria, while the positive values of the PC1 showed a more slender and thin aspect more related with female criteria. The negative values of the PC2 included more characteristic features of free-ranging individuals including a more rounded humeral head and more elongated trochanters compared to captive individuals along the positive values of the PC2 with a greater tubercle more extending medially. Similar variations have also been demonstrated for the distal epiphysis where the negative values of the PC1 and PC2 expressed rather male and free-ranging characters, respectively, with a more marked difference between both subspecies. The trochlea was wider mediolaterally in males and the main axis was more inclined dorsoventrally in females, while it was wider craniocaudally and narrower mediolaterally in captive individuals.

- Radius: Unlike the humerus (proximal and distal parts) and the distal radius epiphysis, the proximal radius epiphysis expressed less sexual variation in the morphospace on the PC1. However, the separation of individual free-ranging and captive reindeer was clearly visible. This results in a massive morphology with large epiphyses along the craniocaudal (positive values of the PC1) and mediolateral (negative values of the PC2) axes in free-ranging individuals. Captive individuals had a more slender morphology with a less extended articular surface laterally, and whose medial and lateral glenoid cavities were less asymmetrical (negative values of the PC1 and positive values of the PC2). Negative values of the PC1 and PC2 indicated a larger distal epiphysis craniocaudally and more extending laterally, further characterizing male and free-ranging individuals, respectively. In captive individuals, the more slender morphology is characterized by a more compressed epiphysis along the craniocaudal axis and the articular surfaces were more extensive along the mediolateral axis.

- Ulna: The proximal part of the ulna showed lifestyle discrimination along the PC1 while the sexual variation was expressed through the PC2. In free-ranging individuals, the cranial edge of the olecranon was thicker than that in captive individuals, and the anconeal process stretched more craniodistally, thus rendering the articular surface constituting the trochlear notch more developed (positive values of the PC1). The negative values of the PC2 exhibited more male features including a more massive olecranon than in females.

- Metacarpal: The proximal part did not show any real distinctions between groups. The negative values of the PC1 nevertheless expressed a more slender theoretical morphology were narrower craniodorsally and elongated mediolaterally. This slender feature seemed to be found more often in free-ranging female *R.t. fennicus* than in male *R.t. tarandus*. The distal epiphysis of the metacarpal, however, showed a good distinction between groups, firstly distributing *R.t. tarandus* and males on the negative values of the PC1, and *R.t. fennicus* and females on the positive values of the PC1. The epiphysis was more compressed proximodistally and more stretched craniocaudally in females and *R.t. fennicus*, while they were more stretched proximodistally and less craniocaudally in males and *R.t. tarandus*. Along positive values of the PC2, further characterizing captive individuals, the medial and lateral edges of the articular eminences widened more along the mediolateral axis.

### Allometry

**Fig. 7:**
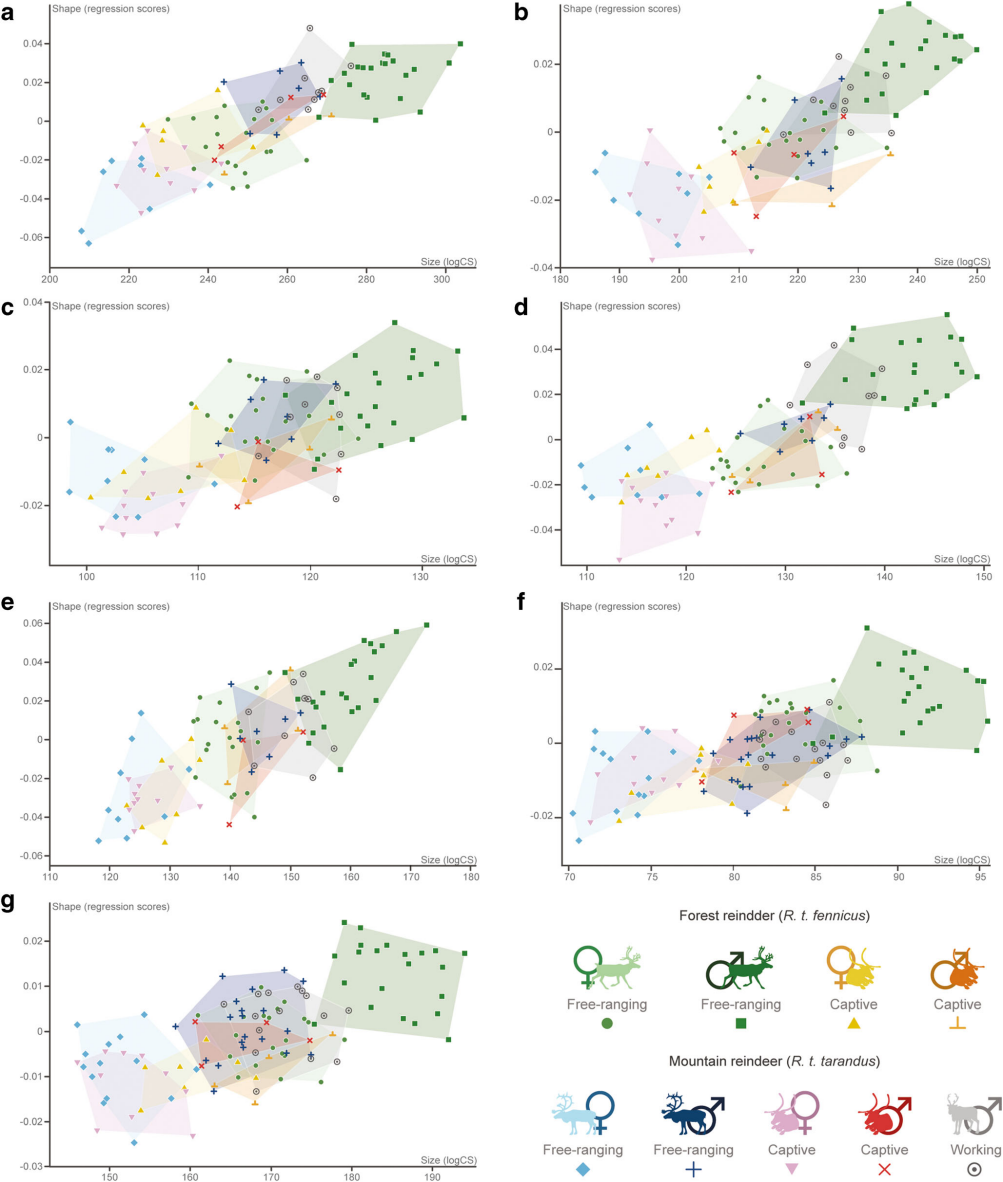
Multivariate regression plots performed on shape data (regression scores) and log-transformed centroid size (logCS) according the subspecies (*R.t. tarandus* and *R.t. fennicus*), sex (male = ♂ and female = ♀) and lifestyle (free-ranging, captive and working). (A) Proximal humerus; (B) distal humerus; (C) proximal radius; (D) distal radius; (E) proximal ulna; (F) proximal metacarpal; (G) distal metacarpal

For all elements, allometry was significant (all *P* < 0.01). Although the percentage of shape variance related to size was always relatively low (proximal humerus, 10.32%; distal humerus, 8.08%; proximal radius, 3.48%; distal radius, 9.60%; proximal ulna, 11.58%; proximal metacarpal, 2.65%; distal metacarpal, 3.40%), this also indicates that the allometric pattern varies slightly depending on the bone. Multivariate regressions of shape scores against log-transformed centroid size display a good discrimination between free-ranging males *R.t. fennicus* with the biggest centroid size on the one hand, and captive and free-ranging female *R.t. tarandus* and captive female *R.t. fennicus* on the other hand (Fig. 7). The separation between these groups was similar and consistent with previous analyses (i.e. size and shape), although controlling the allometry, morphological variations between the intermediate groups was less evident. The overlaps mainly concerned male *R.t. tarandus*, for all lifestyles combined (i.e. captive, free-ranging and working), and free-ranging female *R.t. fennicus*.

## Discussion

### Identifying subspecies in Eurasian reindeer

The interpretation of fossil reindeer bone finds from the Eurasian archaeological context can be complicated due to the presence of two interbreeding subspecies: the mountain reindeer, *R.t. tarandus*, which includes both wild and semi-domesticated herds, as well as the wild forest reindeer, *R.t. fennicus*. Although in some regions of Siberia and Fennoscandia the contact area between the two subspecies is limited, it nevertheless remains very broad across most of Eurasia (cf. Fig. 1b) and was even greater in the past. In addition, morphologies and body proportions of these two subspecies overlap extensively (Nieminen and Helle 1980). Their identification from the dimensions of the postcranial bones is possible but difficult based on fragmented archaeological material (Puputti and Niskanen 2009). Subspecies identification, however, is of great interest to archaeologists in terms of understanding the history of many past Arctic communities, because it can reflect different subsistence strategies (hunting or husbandry) and/or cultural interpretations.

In our study, the centroid-size variations of the different bones analysed allowed us to highlight a significantly larger size in *R.t. fennicus* than in *R.t. tarandus* (whether or not the sex of individuals is included), which is in agreement with previous studies from linear measurements of the postcranial skeleton (Puputti and Niskanen 2009). However, analyses of morphological variation brought new clues of subspecific discrimination. Our results allow a relatively good distinction between the two reindeer subspecies currently living in Fennoscandia, although this varies more or less depending on the bone considered. It would seem that this distinction is even more evident on the distal epiphyses of the forelimb long bones than on the proximal epiphyses. The congruence of these morphotypes with phylogeny indicates that the phylogenetic signal on the shape of long bones is relatively strong.

The morphological differences between subspecies could reflect both behavioural and ecological differences; the distribution of ecotypes is gradually organized according to a North-South geographical line delimited by the distribution of the tundra in the north and the taiga in the south. *R.t. tarandus* is a more gregarious animal, inhabiting in open areas, while *R.t. fennicus* has a more complex social organization in a more closed environment. The withers height of the latter is 10 to 15 cm higher than that of *R.t. tarandus*, mainly due to leg length. This reflects an important adaption to taiga conditions with a deep and soft snow cover, while *R.t. tarandus* lives on hard-packed tundra snow (Nieminen and Helle 1980). However, the two reindeer subspecies share ecologies with significant similarities in some regions, making it difficult to accurately assess the environmental effect on bone shape. In addition, this should be assessed with the utmost caution since over time, it is historically known that the ranges of wild and domestic reindeer have fluctuated greatly under the pressure of various anthropogenic and/or climatic factors (Ingold 1980; Reimers and Colman 2006; Pape and Löffler 2012; Bergman et al. 2013).

Despite the complex interaction of intrinsic and/or extrinsic factors that can infer the morphology of current subspecies, our results concerning both size and shape can help distinguish them from the fossil record. The cross-validated classification results were also generally better (> 80%) for the humerus and metacarpal. Knowing that *R.t. tarandus* presents a (semi-)domestic form in most arctic regions and *R.t. fennicus* only its wild form, the identification of the subspecies seems to be an essential prerequisite before any identification of domestic individuals. Among certain ethnic groups such as Sámis, Nganasans, Evens or Evenks, wild reindeer hunting had long been practiced in parallel with the breeding of domestic reindeer herds, which reveals deep cultural differences and subsistence strategies (Popov 1966; Baskin 2000; Ryd 2011; Salmi et al. 2018), especially in regions where the geographic distribution of the two subspecies largely overlaps.

### Implication of sexual dimorphism

Phylogeny is not the only or main effect causing variations in the shape of the bony elements of the forelimbs. Indeed, a large part of the observed morphological variation between groups is associated with the sex of the individuals. Our results indicated that males had significantly larger bone elements than females. This can be explained in particular by a strong sexual dimorphism in reindeer (Klein 1970; Reimers et al. 1983; Weinstock 2000; Puputti and Niskanen 2009; Melnycky et al. 2013). This difference was also significant within both subspecies, where male *R.t. fennicus* were the largest, female *R.t. tarandus* were the smallest and finally male *R.t. tarandus* were slightly larger than female *R.t. fennicus*. Generally, this results in a more massive morphology with larger epiphyses in males, while females have a thinner and slender morphology.

Although cross-validated classification varied between the different elements, it had better results for correct sex attribution than for subspecies identification, with, in particular, a correct classification percentage between 83 and 92% for proximal and distal humerus, distal radius, proximal ulna and distal metacarpal epiphyses. These morphological differences between sexes are probably due to the different weight-bearing functions of the skeletal elements. The long bones of the forelimbs carry a greater share of the body weight, but also the weight of antlers, which are bigger and heavier in males. This biological characteristic would explain in part why the long bones of the forelimb are good sex discriminators in reindeer (Weinstock 2000; Puputti and Niskanen 2009).

The identification of sex is relatively easy and often applied in archaeology to establish sex ratios and identify the hunting methods and/or the reindeer exploitation by past human societies (e.g. Weinstock 2000; Puputti and Niskanen 2009; Salmi et al. 2015). Our results showed that in addition to allowing identification of subspecies from the proximal and distal elements of the forelimbs, it is also possible to correctly identify the sex, using size and/or shape data. The identification of sex has an important implication in order to find domestic individuals in the fossil record. In Fennoscandia, for example, the Sámis have long kept their nomadic traditions and their ancestral wild reindeer hunting practices. They used only small domestic reindeer herds as decoys for hunting wild individuals, and some female reindeer were also used for milking (Tegengren 1952; Aronsson 1991). Some domestic individuals, especially castrated males, were used to perform various domestic tasks, such as pulling sleds and carrying freight (Korhonen 2008). Among the Evenks (Southeastern Russia) or Tsaatans (Northern Mongolia), castrated males were also used for riding (Mirov 1945; Inamura 2005; Anderson et al. 2017). Among the Komi herders (Northwestern Russia), castrated males are used for transport, kept close to dwellings and separated from the rest of the herd composed of females and uncastrated males (Dwyer and Istomin 2008). Although castrated individuals could not yet be included in our study, the identification of these particular males in the fossil record could also be an excellent proxy for human management of reindeer populations and will have to be tested in the future.

In Eurasia, despite the great variability in husbandry practices among different groups of indigenous reindeer herders, a balance can generally be observed between the sexes within domestic herds. Within the different ethnic groups, most of them still nomadic populations, there is a recurrence to preferentially use domestic males for transport, pulling, riding or racing. Indeed, males, being larger and more robust than females, are more apt to carry out domestic tasks. Among some groups of reindeer herders, domestic female individuals may also be used for milking. According to the historical and or geographic context, as well as the indigenous people studied, sex is just like the subspecies, essential to recognize in order to identify domestic individuals and the type of husbandry from archaeological material.

### Influence of lifestyle and impact of captivity on reindeer morphology

Phylogeny, ecology and sex do not alone explain all the morphometric variation observed within our sample. Indeed, lifestyle also has a significant impact on the size and morphology of the individuals composing the different groups of reindeer (male or female; forest or mountain reindeer). In general, the bony elements of individuals bred in captivity experienced a decrease in their centroid size in comparison with free-ranging individuals. The reduction in body size affecting captive individuals is a typical characteristic generally used to document the effects of domestication (Davis 1981; Payne and Bull 1988; Morey 1992; Dayan 1994; Zohary et al. 1998; Peters et al. 1999; Albarella et al. 2005; Zeder et al. 2006; Hongo et al. 2009). Despite the small number of captive individuals in our sample, this rule seems particularly well respected for wild forest reindeer (*R.t. fennicus*). Zoo individuals in our study do not have a long ancestry in the zoo; most of the Linnanmaa reindeer were born in the wild (Pudas, *personal communication*). Therefore, the effects of reduced mobility are evident without preferential selection. However, this is less evident in *R.t. tarandus*. This could be partly explained by the fact that in Finland, following introgression of domestic reindeer into the wild gene pool in the 19th century, there are no longer any completely wild modern *R.t. tarandus* genetic lineages (Røed et al. 2011, 2014). On the other hand, this could also be the result of selective breeding in captivity. Thus, the impact of captivity on these individuals was not perceptible in our small sample, but could potentially have occurred during the early stages of domestication.

In terms of size and shape, we did not detect any significant difference between free-ranging and captive female *R.t. tarandus*, but on the other hand they differed relatively well from the other categories. Female *R.t. tarandus*, whose bony elements were the smallest, were characterized by a generally slenderer morphology than male *R.t. tarandus* or *R.t. fennicus*. On the contrary, our results showed that male *R.t. fennicus* were also relatively easy to identify based on size (larger) and shape (more robust). Thus, *R.t. tarandus* females and *R.t. fennicus* males are therefore potentially the easiest to identify or exclude from archaeological material, which has opposite socio-economic implications (husbandry versus hunting) as well as a different human-reindeer relationship.

Issues concerning male *R.t. tarandus* are more debatable. In our study, we could not prove the decrease in size due to the effect of captivity, as was the case in male *R.t. fennicus*, and thus facilitate the identification of domestic individuals. No significant difference was found between free-ranging, captives and working individuals, although working individuals tended to be slightly larger than free-ranging individuals. This could be explained by the fact that working individuals are selected for their physical properties and abilities. Size reduction should therefore not be a reliable criterion for identifying male domestic *R.t. tarandus* kept in captivity. The absence of a significant difference in shape of the bony elements between free-ranging and working *R.t. tarandus* could be explained by the fact that, apart from their training in running, which only takes place during the winter, working individuals were left free-range the rest of the year, just as free-ranging individuals. Activity levels can therefore be similar most of the year, leading to similar size and robustness requirements. In addition, forelimb long bones are mainly adapted to support body mass, while hindlimb bones are more apt for propulsion, and are more impacted by external pressures (McGuigan and Wilson 2003; Hanot et al. 2017). Morphological differences were therefore not observed in our study on the forelimb, but this hypothesis will have to be tested in the future on hindlimb long bones in order to try to understand possible variations between captive, free-ranging or working reindeer. In addition, the weight of the antlers and the body mass of males is therefore a criterion for greater robustness of the forelimbs and is morphometrically very different from females (Weinstock 2000; Puputti and Niskanen 2009). In addition, working males carry loads, the weight of which is mainly borne by the forelimbs of the animal. Although we could not include analysis of such individuals as part of our modern sample, it could be a parameter affecting the bone morphology of male domestic individuals (Shackelford et al. 2013).

The robustness of the bones in the biggest individuals resulted in an overall enlargement of the epiphyses. Captivity, which induces a decrease in activity and body size, could therefore play a direct role in bone shape. For the proximal epiphysis of the humerus, the lesser tubercle was more developed at the expense of the greater tubercle in captive individuals, allowing greater stability of the shoulder and better resistance to adduction of the humerus (Watson and Wilson 2007; Mallet et al. 2019). This is associated with a lengthening of the insertion for the *Subscapularis* muscle, possibly due to increased time spent immobile with the shoulder-bracing muscle apparatus activated (Niinimäki and Salmi 2016). The distal epiphysis of the humerus is more mediolaterally enlarged in free-ranging individuals, increasing the articular surface with the trochlear notch (formed by the proximal radius and ulna, also larger craniocaudally) and provides a greater stability of the elbow joint and larger insertion areas for the different flexor and extensor muscles for the digits (Jenkins 1973; Mallet et al. 2019). This would result from repetitive flexing of the elbow articulation, for instance when digging for lichen under the snow. In the winter, Scandinavian reindeer dig for lichen buried beneath the snow using their forelimbs, while this activity is reduced or even absent in captive individuals, because they were fed by their owners (Niinimäki and Salmi 2016). In addition, the distal radius and distal metacarpal epiphyses appeared to widen mediolaterally among captive individuals. This widening of more caudal limb elements and their distal ends could be a result of the need for strengthening articular areas for prolonged periods of static loading.

The decrease in body size of wild individuals under the effect of captivity could therefore be the first element to consider in identifying domestic individuals in the archaeological record. However, this does not concern all the individuals composing the herds kept by the herders, as most of them are left to free-range. Especially today, free-ranging *R.t. tarandus* are given additional fodder during winter while they live in free-range and can also find their own food, whereas *R.t. fennicus* are not domesticated and thus do not receive additional food. A focus must be on particular individuals, namely some domestic female *R.t. tarandus* which could be used for milking and kept near the living area, or bigger male *R.t. tarandus* which are rather selected for domestic tasks like transport, pulling, riding or racing. In addition, this control of mobility (i.e. captivity) combined with changes in feeding behaviour (i.e. additional food provided by herders) induces significant changes in the bone morphology of reindeer. As the activity of captive individuals is greatly reduced, long bones of the forelimb would trend towards greater gracility than their free-ranging counterparts, both for subspecies and for sex.

### Application perspectives for the identification of early domesticated reindeer in archaeology

To our knowledge, there is no evidence that the Fennoscandian reindeer were kept in total captivity from the Iron Age. These could be small herds kept under fairly close supervision by Sámi herders (from 3–4 to several dozen individuals), sometimes kept corralled close to human settlements, especially because they were used as decoy animals for hunting, and for transport or milking (Itkonen 1948; Tegengren 1952; Helle and Jaakkola 2008; Korhonen 2008; Andersen 2011; Bjørklund 2013). The mobility control of these individuals therefore induced lower levels of physical activity compared to free-ranging animals. Managing individuals in captivity is however observed nowadays in different indigenous groups of Eurasia.

Among the Tsaatans, herds of 7 to 160 individuals are kept very close to their tents, often hitched to stakes or driven into a wooden enclosure (Inamura 2005; Haas et al. 2019). Reindeer are sometimes pastured in pairs with their necks tied to each other with rope in order to impede their movement and keep them from running away (Inamura 2005). Among the Evenks of southern Russia, the herd size is relatively equivalent to the Tsaatans: between a dozen and more than two hundred individuals. However, only castrated male reindeer used for transport or riding are kept in corrals (Anderson et al. 2017). Among the Selkups, reindeer can be kept very close to houses, or even inside houses specially built to protect them from insects (Piezonka et al. 2020). In contrast, among the Nenets and Komi herders, the organization of husbandry is relatively different since the herds can amount to between 1,500 and 4,000 reindeer. Unlike the Nenets who do not separate the individuals, the Komi separate the herds into two distinct groups. The first group of reindeer consists mainly of castrated males used for transport (around 10–20% of individuals) that are always pastured in close proximity to the settlements (i.e. controlled mobility), in order for the nomads to always have a means of transport at their disposal. The second group includes female reindeer, and uncastrated males, as well as calves (which includes 80–90% of the total number of animals). These individuals are generally left to range freely within 10–15 km of the camp (Dwyer and Istomin 2008).

Herding methods can therefore differ completely from region to region or from one ethnic group to another. Given the lifestyle of reindeer past and present, mostly left as free-ranging, research must focus on particular domestic individuals whose mobility were largely controlled by humans. Also, the presence of wild individuals identified from fossil bones, such as wild *R.t. fennicus*, is not necessarily proof of the absence of domestication by an ethnic group. Indeed, many nomadic indigenous peoples have long continued to hunt wild reindeer along with the breeding of domestic individuals (Baskin 2000; Hansen and Olsen 2014; Reindeer Herding 2019). For example, in Sámi sacrificial sites, the deposit of wild and/or domestic reindeer bones was frequent and could reflect cultural changes within these groups (Salmi et al. 2015, 2018, 2020a; Salmi and Heino 2019).

Another factor that can lead to a misidentification of domestic individuals in archaeological assemblages is the possibility of finding hybrid individuals resulting from crossbreeding between wild *R.t. fennicus* and semi-domesticated reindeer. This hybridization between wild individuals from the two subspecies is very common in regions where their distribution ranges overlap, but can also occur between wild and domestic individuals, especially in free-ranging individuals (Nieminen and Helle 1980; Nieminen and Ojutkangas 1986; Røed et al. 2008, 2011). Among the Evenks, reproduction is controlled and herders occasionally allow reproduction between wild males and domestic females (Anderson et al. 2017). In Fennoscandia, it is likely that wild individuals were captured to incorporate them into domestic herds in order to avoid consanguinity (Sommerseth 2011). Although we have not been able to include hybrid individuals in our study, the main problem of their presence in archaeological contexts could be the potential large overlap between hybrids and their parents in terms of morphometric diversity. Indeed, hybrids can present morphological traits more similar to a particular parent, as well as an intermediate morphology and size (Evin et al. 2015; Hanot et al. 2017, 2019; Hanot and Bochaton 2018; Savriama et al. 2018).

## Conclusion

The reindeer is probably one of the most recent species to have been domesticated by humans and is still considered to be in the early stages of the domestication process. Thus, zooarchaeologists need powerful biomarkers on the skeleton to document the origin of this process. Understanding the morphometric variability of reindeer had to be carried out beforehand by bringing together a large sample of modern specimens before application to the fossil record. Our work demonstrated the potential of 3D GMM studies in order to identify both subspecies and sex of free-ranging reindeer or those that live in captivity. Our results showed that size and/or shape of most of the isolated elements of the forelimb allowed a relatively reliable distinction between wild and domestic individuals. This methodology will allow archaeologists to better estimate the presence of wild or domestic reindeer in archaeological assemblages, and thus to comprehend the evolution of socio-economic models of the different Arctic communities of reindeer herders in Eurasia. In addition, this 3D GMM approach allows us to understand the meaning of morphological variation under the effect of reduced mobility (i.e. captivity) or change in feeding behaviour (fed or self-feeding) induced by domestication. It also suggests that our protocol can be adapted for other domesticated ungulate species that have been corralled, fed or used for domestic tasks (racing, riding and pulling), such as bovines, camelids, caprines, equids or suids. However, caution must be taken with regard to correct identification of domestic reindeer due to the great variability of husbandry and the dispersion of the domestication process, as well as the genetic introgression between wild and domestic herds. Each parameter such as size, shape and allometry must absolutely be finely analysed and coupled with archaeological contexts in order to be able to identify individuals and better understand the morphometric variability of reindeer in Eurasia. New studies allowing the better understanding of the morphometric diversity of reindeer should be carried out in order to complement our study on the forelimb (e.g. hindlimb, cross-sections, teeth). Such studies would allow the refinement of research on archaeological sites for better identification of the first stages of domestication in time and space.
